# Supplementary material for: Proton-Detected Solid-State NMR for Deciphering Structural Polymorphism and Dynamic Heterogeneity of Cellular Carbohydrates in Pathogenic Fungi
Source: J Am Chem Soc. 2025 May 6;147(20):17416–32. doi: 10.1021/jacs.5c04054 (PMC12100651; doi:10.1021/jacs.5c04054)
Supplement: Supplementary file 1 [file ja5c04054_si_001.pdf]

Supplementary Information

Proton-Detected Solid-State NMR for Deciphering Structural Polymorphism and Dynamic Heterogeneity of Cellular Carbohydrates in Pathogenic Fungi

Jayasubba Reddy Yarava[1‡]*, Isha Gautam[1‡], Anand Jacob[1], Riqiang Fu[2], Tuo Wang[1]*

[1] Department of Chemistry, Michigan State University, East Lansing, MI 48824, USA

[2] National High Magnetic Field Laboratory, Florida State University, Tallahassee, FL 32310, USA

‡ These authors contributed equally

* Correspondence: yaravaja@msu.edu; wangtuo1@msu.edu

Table of Contents

Text S1. Phase cycling schemes used for the NMR pulse sequences

Text S2. Relaxation rate equations

Text S3. Simple model free (SMF) formalism

Figure S1. 1D and 2D spectra of fungal samples collected at different time points

Figure S2. Proton detection-based pulse sequences for fungal cell wall analysis

Figure S3. Comparison of linewidths of protonated and deuterated *A. fumigatus*

Figure S4. Comparison of 2D hCH spectra of deuterated *A. fumigatus* at different magnetic fields

Figure S5. ^{1}H-^{13}C correlation spectra of the mobile region of *C. albicans*

Figure S6. ^{13}C R_1 decay curves of *R. delemar*

Figure S7. ^{13}C $R_{1\rho}$ decay curves of *R. delemar*

Figure S8. ^{13}C R_1 decay curves of deuterated *A. fumigatus*

Figure S9. ^{13}C $R_{1\rho}$ decay curves of deuterated *A. fumigatus*

Table S1. Experimental parameters used for *R. delemar*

Table S2. Experimental parameters used for deuterated *A. fumigatus* at 600 MHz

Table S3. Experimental parameters used for deuterated *A. fumigatus* at 800 MHz

Table S4. Experimental parameters used for *C. albicans*

Table S5. ^{1}H and ^{13}C chemical shifts of rigid carbohydrates of *R. delemar*

Table S6. ^{1}H and ^{13}C chemical shifts of deuterated *A. fumigatus*

Table S7. ^{1}H and ^{13}C chemical shifts of mobile carbohydrates of *C. albicans*

Table S8. Dynamical parameters of *R. delemar*

Table S9. Dynamical parameters of deuterated *A. fumigatus*

Supplementary references

Text S1. Phase cycling schemes used for the NMR pulse sequences in Figure S1.

a. 2D hCH/2D hNH:

ϕ_1 = +y, -y; ϕ_2 = +x; ϕ_3 = +y; ϕ_4 = +x; ϕ_5 = +x; ϕ_6 = +x, +x, -x, -x; ϕ_7 = +y; ϕ_8=+y, +y, +y, +y, -y, -y, -y, -y; ϕ_{rec} = +y, -y, -y, +y, -y, +y, +y, -y;

b. 3D hcoCH$_3$coNH:

ϕ_1 = +y, -y; ϕ_2 = +x ; ϕ_3 = +y, +y, -y, -y; ϕ_4 = +x ; ϕ_5 = +x ; ϕ_6 = +x; ϕ_7 = +y; ϕ_8 = +y; ϕ_9 = +y, +y, +y, +y, -y, -y, -y, -y; ϕ_{10} = +x, +x, +x, +x, +x, +x, +x, +x, -x, -x, -x, -x, -x, -x, -x, -x; ϕ_{11} = -y ; ϕ_{12} = +y; ϕ_{13} = +x; ϕ_{14} = +x; ϕ_{rec} = +x, -x, -x, +x, -x, +x, +x, -x, -x, +x, +x, -x, +x, -x, -x, +x;

c. 3D hc2NH:

ϕ_1 = +x, -x; ϕ_2 = +y; ϕ_3 = +x; ϕ_4 = +x; ϕ_5 = +x; ϕ_6 = +y; ϕ_7 = +x, -x; ϕ_8 = +x; ϕ_9 = +x, +x, -x, -x; ϕ_{10} = +y; ϕ_{11} = +y, +y, +y, +y, -y, -y, -y, -y; ϕ_{rec} = +y, -y, -y, +y, -y, +y, +y, -y;

d. 3D hCHhH (RFDR):

ϕ_1 = +y, -y; ϕ_2 = +x; ϕ_3 = +y; ϕ_4 = +x ; ϕ_5 = +x; ϕ_6 = +x, +x, -x, -x; ϕ_7 = +y; ϕ_8 = +y ; ϕ_9 = -x; ϕ_{10} = +x, +x, +x, +x, +y, +y, +y, +y, -x, -x, -x, -x, -y, -y, -y, -y; (RFDR-XY8) = +x, +y, +x, +y, +y, -x, +y, -x, -x, -y, -x, -y, -y, +x, -y, +x; ϕ_{rec} = +x, -x, -x, +x, +y, -y, -y, +y, -x, +x, +x, -x, -y, +y, +y, -y;

e. 3D hCCH TOCSY (WALTZ-16)

ϕ_1 = +y, +y, -y, -y; ϕ_2 = +x; ϕ_3 = +x, -x; ϕ_4 = +x; ϕ_5 = +y; ϕ_6 = +x, +x, +x, +x, -x, -x, -x, -x; ϕ_7 = -x; ϕ_8 = +y; ϕ_9 = +x; ϕ_{10} = +x; ϕ_{rec} = +x, -x, -x, +x;

f. 2D ^{1}H-^{13}C T_1 hCH

ϕ_1 = +y, -y; ϕ_2 = +x; ϕ_3 = +y; ϕ_4= +x; ϕ_5 = +x; ϕ_6 = -x, -x, -x, -x, -x, -x, -x, -x, +x, +x, +x, +x, +x, +x, +x, +x; ϕ_7 = +x; ϕ_8 = -x, -x, +x, +x; ϕ_9 = +y, +y, +y, +y, -y, -y, -y, -y; ϕ_{10} = +y; ϕ_{rec} = +y, -y, -y, +y, -y, +y, +y, -y, -y, +y, +y, -y, +y, -y, -y, +y

g. 2D ^{1}H-^{13}C $T_{1\rho}$ hCH

ϕ_1 = +y, -y; ϕ_2 = +x; ϕ_3 = +y; ϕ_4 = +x; ϕ_5 = +y; ϕ_6 = x; ϕ_7 = +x, +x, -x, -x; ϕ_8 = +y; ϕ_9 = +y, +y, +y, +y, -y, -y, -y, -y; ϕ_{rec}= +y, -y, -y, +y, -y, +y, +y, -y

h. 2D ^{1}H-^{13}C *J*-INEPT-HSQC

ϕ_1 = +x, +x, +x, +x, -x, -x, -x, -x; ϕ_2 = +x; ϕ_3 = +x; ϕ_4 = +y; ϕ_5 = +x, -x; ϕ_6 = +x; ϕ_7 = +x; ϕ_8 = +y; ϕ_9 = -y; ϕ_{10} = +x; ϕ_{11} = +x; ϕ_{12} = +x; ϕ_{13} = +x, +x, -x, -x; ϕ_{14} = +x; ϕ_{15} = +x; ϕ_{16} = +x; ϕ_{rec} = +x, -x, -x, +x, -x, +x, +x, -x

i. 3D ^{1}H-^{13}C *J*-CCH-TOCSY (DIPSI-3)

ϕ_1 = +x; ϕ_2 = +x; ϕ_3 = +x; ϕ_4 = -y; ϕ_5 = +x, -x; ϕ_6 = +x; ϕ_7 = +x; ϕ_8 = +y; ϕ_9 = -y, -y, +y, +y; ϕ_{10} = +x; ϕ_{11} = +x; ϕ_{12} = +x, +x, +x, +x, -x, -x, -x, -x; ϕ_{13} = -x; ϕ_{14} = +x, -x; ϕ_{15} = +x; ϕ_{rec} = +x, -x, -x, +x, -x, +x, +x, -x

j. 1D ^{1}H-^{13}C T_1 filter CP

ϕ_1 =+y, -y; ϕ_2 =+x; ϕ_3 =+x, +x, +y, +y, -x, -x, -y, -y; ϕ_4 =+y, +y, -x, -x, -y, -y, +x, +x ; ϕ_5 =-y, -y, +x, +x, +y, +y ,-x, -x ; ϕ_{rec} =+x, -x, +y, -y, -x, +x, -y, +y

k. 1D ^{1}H-^{13}C T_1 dipolar-dephasing CP

ϕ_1 = +y, -y; ϕ_2 = +x; ϕ_3 = +x, +x, -x, -x, +y, +y, -y, -y; ϕ_4 = +x, +x, -x, -x, +y, +y, -y, -y; ϕ_{rec} = +x, -x, -x, +x, +y, -y, -y, +y

l. 2D ^{1}H $T_{1\rho}$ filtered ^{1}H-^{15}N HETCOR

ϕ_1 =+y, -y; ϕ_2 = +x; ϕ_3 = +x, +x, -x, -x; ϕ_4 = +x; ϕ_5 = +x, +x, +x, +x, -x, -x, -x, -x, +y, +y, +y, +y, -y, -y, -y, -y; θ_M = +y; $-\theta_M$ = -y; FSLG = +x, -x; ϕ_{rec} = +x, -x, +x, -x, -x, +x, -x, +x, +y, -y, +y, -y, -y, +y, -y, +y

Text S2. Relaxation rate equations

Spin relaxation is influenced by the various nuclear spin and spin-spin interactions. For this work, we followed the relaxation rate equations described by Lamely et al.[1], Yarava et al.[2] Busi et al.[3]. For 13C R1 and R1ρ, the relaxation mechanisms with that influence from 13C-1H heteronuclear dipolar couplings and 13C chemical shift anisotropy (CSA). Since these samples are uniformly doubly labeled (13C-and 15N), there are additional mechanisms arising from homonuclear 13C-13C and heteronuclear 13C-15N dipolar couplings. Additioanlly the the 13C R1ρ relaxation rates are also influenced by the spin-lock frequency and MAS rate, all of which are accounted for in the analysis and are described below.

Text S2.1. Spin-lattice relaxation rate.

(a) Impact of CSA on the ^{13}C R_1 relaxation rate

$$R_{1,C,CSA} = \frac{2}{15}\omega_C^2(\Delta\sigma^2)(J_1(\omega_C) \tag{1}$$

Note: For the ^{13}C CSA we employed $\Delta\sigma$~40-80 ppm[4]

(b) Impact of ^{13}C-^{13}C dipolar interaction on the ^{13}C R_1 relaxation rate

$$R_{1,C1C2} = \frac{1}{10}\left(\frac{\mu_0}{4\pi}\frac{\hbar\gamma_C\gamma_C}{r_{CC}^3}\right)^2(J_0(\omega_{C1}-\omega_{C2}) + 3J_1(\omega_{C1}) + 6J_2(\omega_{C1}+\omega_{C2})) \tag{2}$$

Note: $J_0(\omega_{C1}-\omega_{C2})$ was set to 45 ppm for ^{13}C. The distance r_{cc} = 1.525 A^o.

(c) Impact of ^{1}H-^{13}C dipolar interaction on the ^{13}C R_1 relaxation rate

$$R_{1,CH} = \frac{1}{10}\left(\frac{\mu_0}{4\pi}\frac{\hbar\gamma_C\gamma_H}{r_{CH}^3}\right)^2(J_0(\omega_H-\omega_C) + 3J_1(\omega_C) + 6J_2(\omega_H+\omega_C)) \tag{3}$$

Note: The sample is fully protonated, and we have also considered the contribution from remote proton with the distance of 1.8 A^o. The directly bonded ^{13}C to ^{1}H distance is set to r_{CH} = 1.114 A^o.

(d) Impact of ^{13}C-^{15}N dipolar interaction on the ^{13}C R_1 relaxation rate

$$R_{1,CN} = \frac{1}{10}\left(\frac{\mu_0}{4\pi}\frac{\hbar\gamma_C\gamma_N}{r_{CN}^3}\right)^2(J_0(\omega_C-\omega_N) + 3J_1(\omega_C) + 6J_2(\omega_C+\omega_N)) \tag{4}$$

Note: The distance r_{CN} = 1.46 A^o.

Text S2.2. Spin-lattice relaxation rate in the rotating frame.

(e) Impact of CSA on the ^{13}C' $R_{1\rho}$ relaxation rate

$R_{1\rho,C,CSA} = \frac{1}{45}\omega_C^2(\Delta\sigma^2)(\frac{2}{3}J_0(\omega_1 + 2\omega_r) + \frac{2}{3}J_0(\omega_1 - 2\omega_r) + \frac{4}{3}J_0(\omega_1 + \omega_r) + \frac{4}{3}J_0(\omega_1 - \omega_r) + 3J_1(\omega_C)$ (5)

Note: For the ^{13}C CSA we employed $\Delta\sigma = 100$ ppm.

(f) Impact of ^{13}C-^{13}C dipolar interaction on the ^{13}C $R_{1\rho}$ relaxation rate

$R_{1\rho,C1C2} = \frac{1}{20}\left(\frac{\mu_0}{4\pi}\frac{\hbar\gamma_C\gamma_C}{r_{C1C2}^3}\right)^2 (\frac{2}{3}J_0(\omega_1 + 2\omega_r) + \frac{2}{3}J_0(\omega_1 - 2\omega_r) + \frac{4}{3}J_0(\omega_1 + \omega_r) + \frac{4}{3}J_0(\omega_1 - \omega_r) + J_0(\omega_{C1} - \omega_{C2}) + 9J_1(\omega_C) + 6J_2(2\omega_C))$ (6)

Note: $J_0(\omega_{C1} - \omega_{C2})$ was evaluated at a frequency corresponding to 45 ppm for ^{13}C. The distance r_{cc}=1.525 A°.

(e) Impact of ^{1}H-^{13}C dipolar interaction on the ^{13}C $R_{1\rho}$ relaxation rate

$R_{1\rho,CH} = \frac{1}{20}\left(\frac{\mu_0}{4\pi}\frac{\hbar\gamma_H\gamma_C}{r_{CH}^3}\right)^2 (\frac{2}{3}J_0(\omega_1 + 2\omega_r) + \frac{2}{3}J_0(\omega_1 - 2\omega_r) + \frac{4}{3}J_0(\omega_1 + \omega_r) + \frac{4}{3}J_0(\omega_1 - \omega_r) + 3J_1(\omega_C) + J_0(\omega_H - \omega_C) + 6J_1(\omega_H) + 6J_2(\omega_H + \omega_C))$ (7)

The distance r_{CH}=1.114 A°.

(g) Impact of ^{13}C'-^{15}N dipolar interactions on the ^{13}C' $R_{1\rho}$ relaxation rate

$R_{1\rho,CN} = \frac{1}{20}\left(\frac{\mu_0}{4\pi}\frac{\hbar\gamma_C\gamma_N}{r_{CN}^3}\right)^2 (\frac{2}{3}J_0(\omega_1 + 2\omega_r) + \frac{2}{3}J_0(\omega_1 - 2\omega_r) + \frac{4}{3}J_0(\omega_1 + \omega_r) + \frac{4}{3}J_0(\omega_1 - \omega_r) + 3J_1(\omega_C) + J_0(\omega_C - \omega_N) + 6J_1(\omega_N) + 6J_2(\omega_H + \omega_N))$ (8)

The distance r_{CN} = 1.46 A°.

Here μ_0 is the magnetic permeability in vacuum, γ the gyromagnetic ratio for the specified nucleus and $\hbar$ is Planck's constant and *r* is nuclear separation and $J(\omega)$ is the spectral density function.

Text S3. Simple model free (SMF) formalism.

The spectral density is a function of the order parameter (S^2) and effective correlation time τ_{eff}.

$$J(\omega) = (1 - S^2)\frac{\tau_{eff}}{1+(\omega\tau_{eff})^2} \tag{9}$$

The ^{13}C R_1 and $R_{1\rho}$ relaxation data were analyzed with the simple model free formalism[5-6].

The experimental decay intensities were fitted using Mante-Carlo simulation.

The following equation was used to determine the calculated intensities:

$$I_{Rm}^{calc} = I_{Rm}\exp(-R_m(S^2, \tau_{eff},\ \omega)t_k) \tag{10}$$

The experimental and calculated intensities were matched through the minimization of the χ2 function:

$$\chi^2 = \frac{1}{N}\Sigma_{k=1}^{N}\left(\frac{I_{13CR1}^{expt}(t_k) - I_{13CR1}^{calc}(t_k)}{\sigma_{13CR1,expt}^2}\right)^2 + \frac{1}{N}\Sigma_{k=1}^{N}\left(\frac{I_{13CR1\rho}^{expt}(t_k) - I_{13CR1\rho}^{calc}(t_k)}{\sigma_{13CR1\rho,expt}^2}\right)^2 \tag{11}$$

Here, σ_{expt}^2 is the experimental noise and N is the number of experimental relaxation data points.

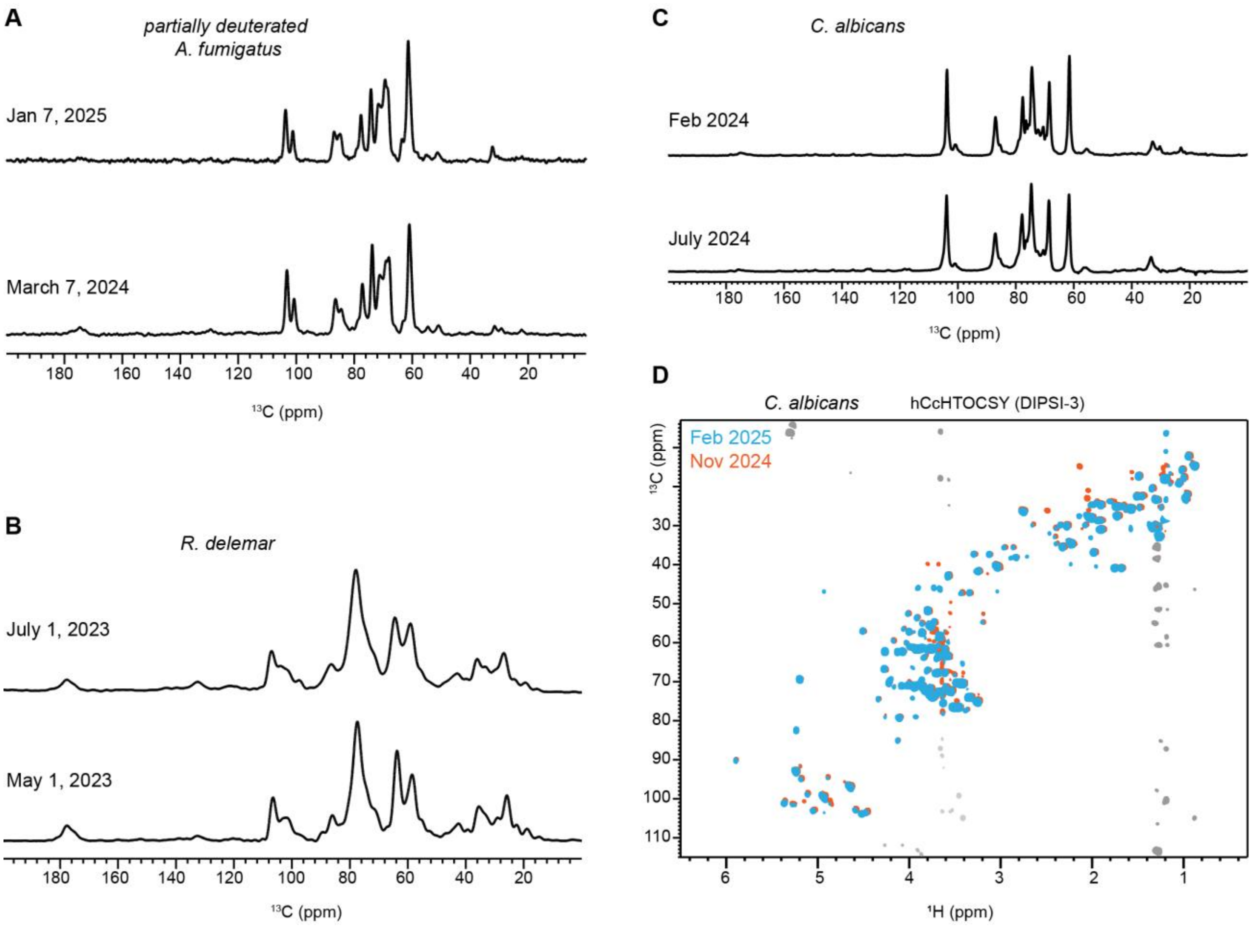

Figure S1. 1D and 2D spectra of fungal samples collected at different time points. 1D ^{13}C spectra of (**A**) partially deuterated *A. fumigatus*, (**B**) *R. delemar*, and (**C**) *C. albicans* measured at different time points, showing consistent spectral patterns within each sample. (**D**) Overlay of two hCcH TOCSY DIPSI-3 spectra collected on *C. albicans* cells at different time points, showing spectral reproducibility.

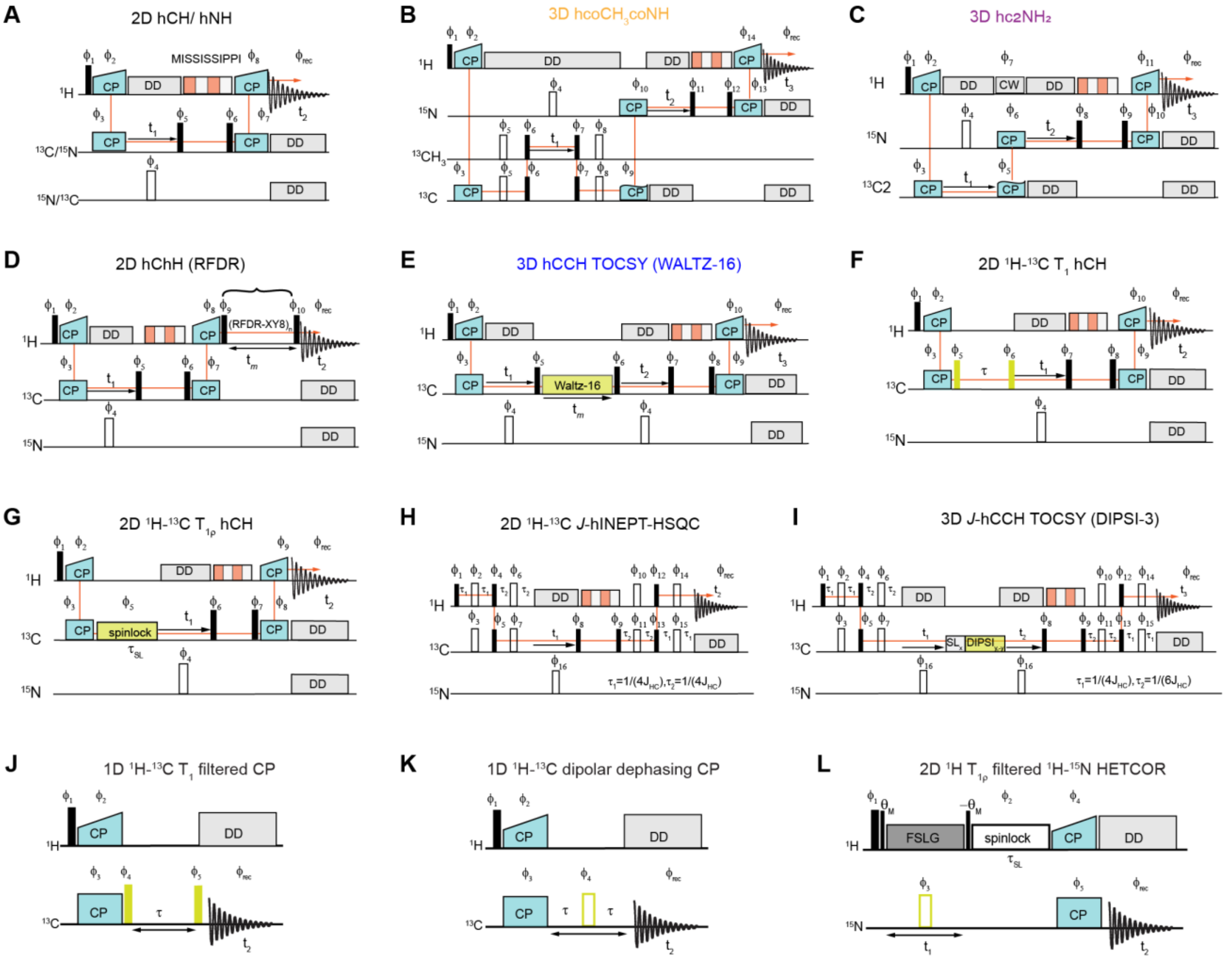

Figure S2. Proton detection-based pulse sequences for fungal cell wall analysis. 2D/3D pulse sequences were used to analyze the rigid and mobile region of the fungal cell wall. (**A**) The 2D hCH and 2D hNH pulse sequences were applied to identify short-range ^1H to $^{13}C/^{15}N$ correlations with water suppression via MISSISSIPPI. In the representative pulse sequences, black rectangles denote $\pi/2$ pulses while open rectangles indicate π pulses. (**B**) The 3D hcoCH3coNH pulse sequence selectively detects chitin by exploiting a unique NH-CO-CH_3 coherence transfer pathway. Magnetization transfer between CO and CH_3 carbons occurs through homonuclear scalar couplings, while heteronuclear dipolar couplings facilitate transfer among ^1H, ^{13}C, and ^{15}N. (**C**) The 3D hc2NH$_2$ sequence selectively detects chitosan via C2-NH_2-H coherence transfer pathway (**D**) The 2D ^1H-^{13}C hChH RFDR pulse sequence established through-space correlations between polysaccharides by employing ^1H-^1H homonuclear dipolar couplings. (**E**) The 3D hCCH TOCSY with WALTZ-16 mixing pulse sequence is used to map through-bond carbon connectivity within polysaccharide components, relying on scalar couplings among ^{13}C nuclei, the TOCSY with WALTZ-16 mixing is highlighted in yellow. (**F, G**) ^{13}C T_1 and ^{13}C $T_{1\rho}$ relaxation times were measured using the 2D ^{13}C T_1 hCH pulse sequence. In the figure, $T_{1,}$ and $T_{1\rho}$ relaxation blocks are highlighted in yellow. (**H**) The highly mobile regions of the cell wall were characterized using the 2D ^1H-^{13}C refocused *J*-INPET-HSQC sequence. (**I**) 3D hCCH TOCSY with DIPSI-3 mixing sequence is applied to establish through-bond carbon connectivity in the mobile regions of the cell wall. The resonances originating from rigid and semi-rigid regions of the cell wall were characterized using relaxation filter pulse sequences (**J**) ^{13}C T_1 filter CP (K) ^{13}C dipolar dephasing CP (**L**) ^1H $T_{1\rho}$ filtered ^1H-^{15}N HETCOR.

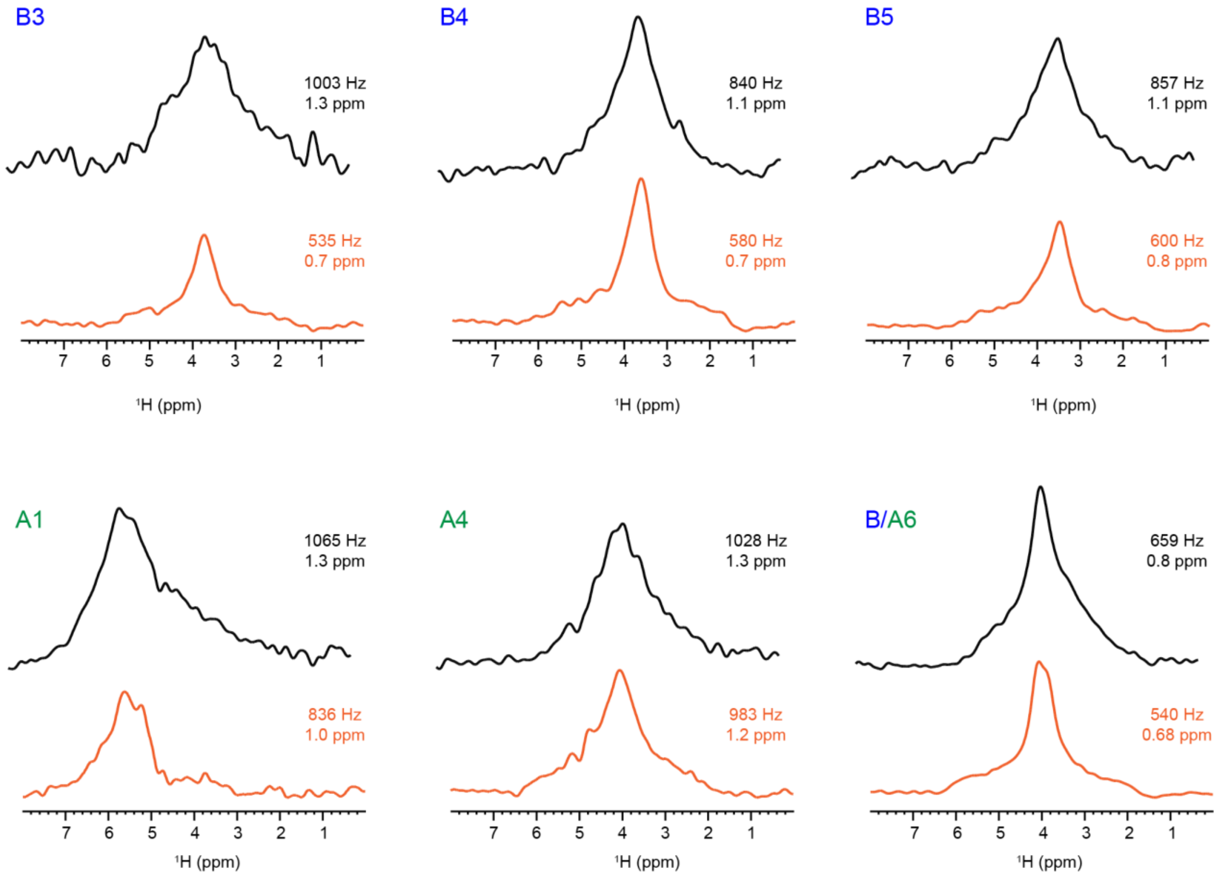

Figure S3. Comparison of linewidths of protonated and deuterated *A. fumigatus* cell wall components. 1D slices were extracted at each carbon site from the 2D hCH correlation spectrum, protonated (black) and deuterated (orange). Spectra were measured on 18.8 Tesla (800 MHz) spectrometer with a MAS rate of 40 kHz. The full width at half height (FWHH) of 1D slice was displayed in the figure. The carbon sites at which the slices were extracted are labelled for β-glucans (B) and α-glucans (A) carbohydrates.

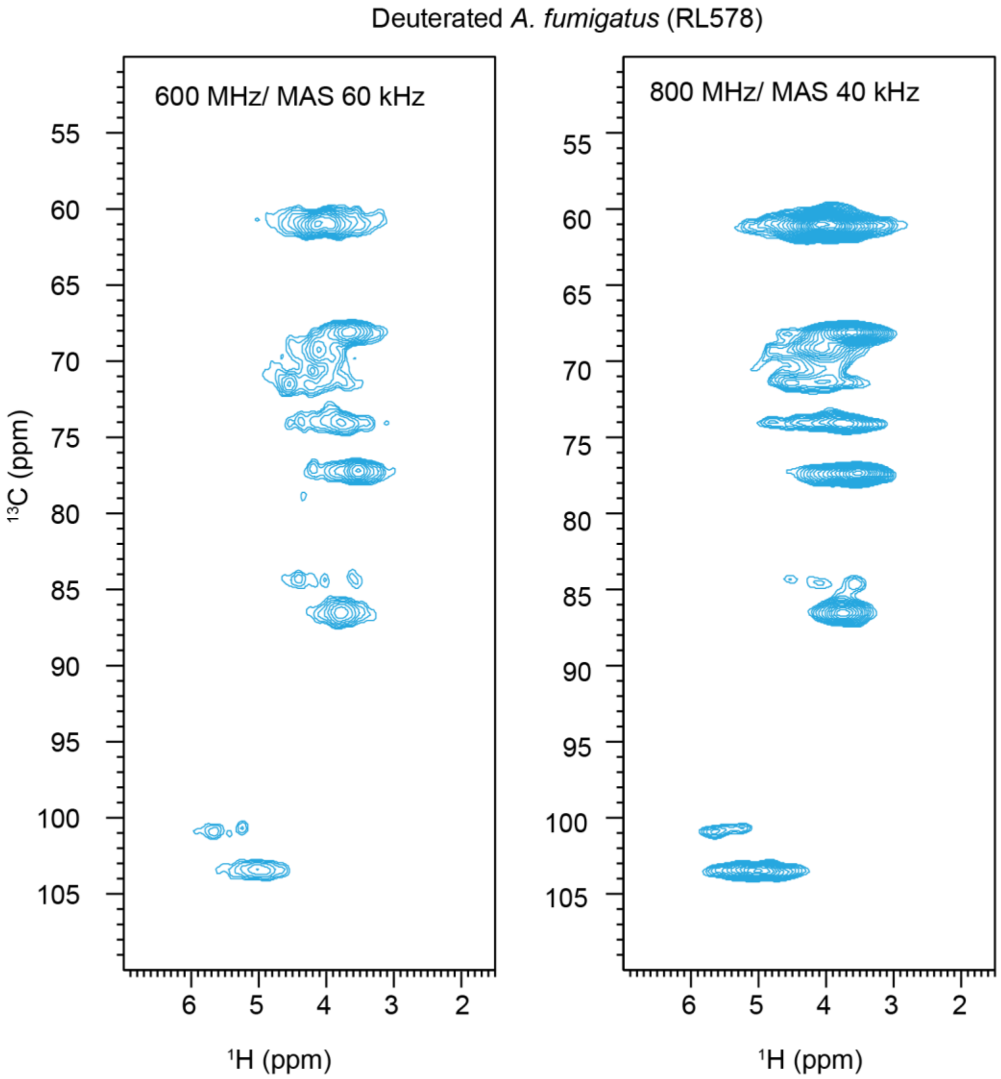

Figure S4. Comparison of deuterated *A. fumigatus* (RL578) spectra at two magnetic fields. Comparing 2d hCH spectra of *A. fumigatus* measured on 14.1 T (600 MHz) spectrometer at MAS rate of 60 kHz (Left) to the spectra measured on 800 MHz (18.8 T) with MAS rate of 40 kHz (Right). The second CP contact time was set to 50 µs for detecting carbon directly attached protons.

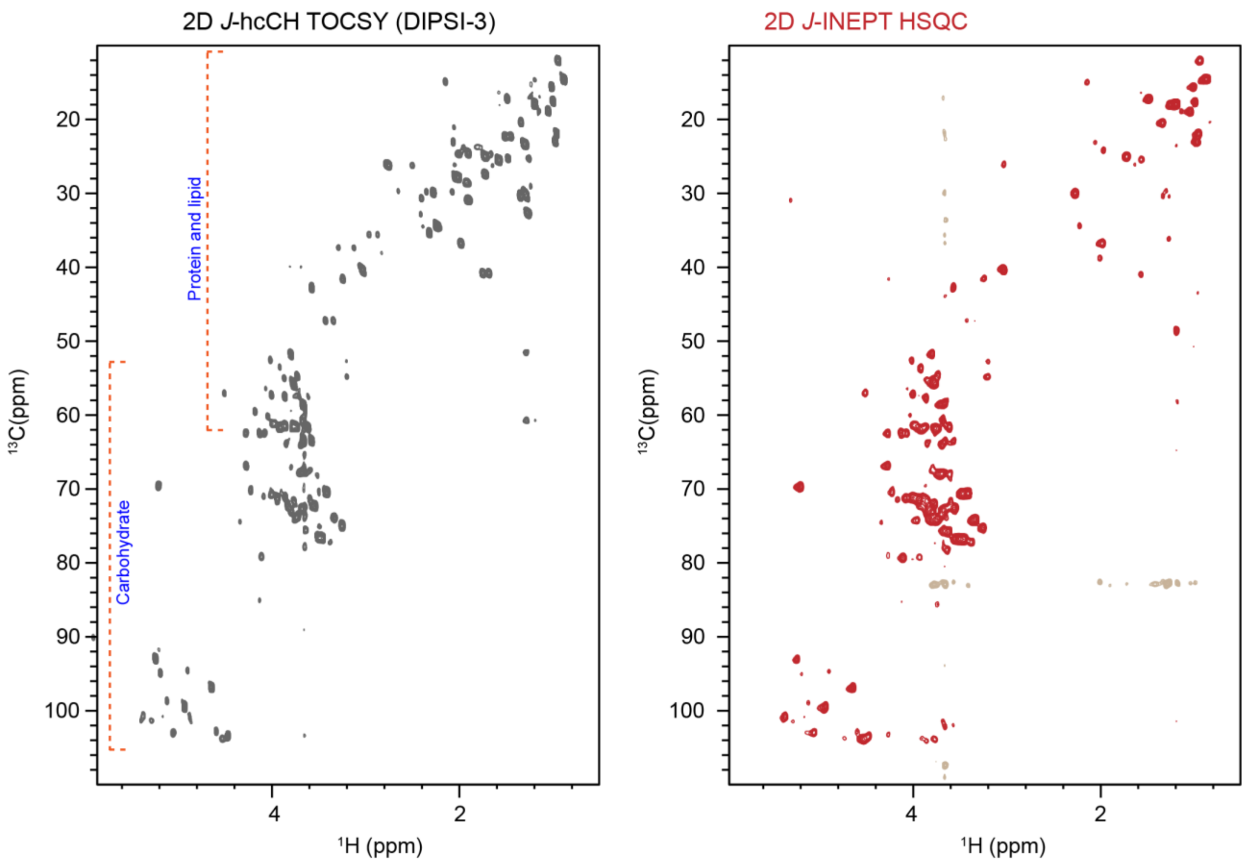

Figure S5. ^{1}H-^{13}C correlation spectra of the mobile region of *C. albicans* (JKC2830). The 2D J-hcCH TOCSY (DIPSI-3) spectra in grey and the 2D ^{1}H-^{13}C J-INEPT HSQC in red of *C. albicans* (JKC2830) acquired on an 18.8 T spectrometer at a MAS rate of 15 kHz.

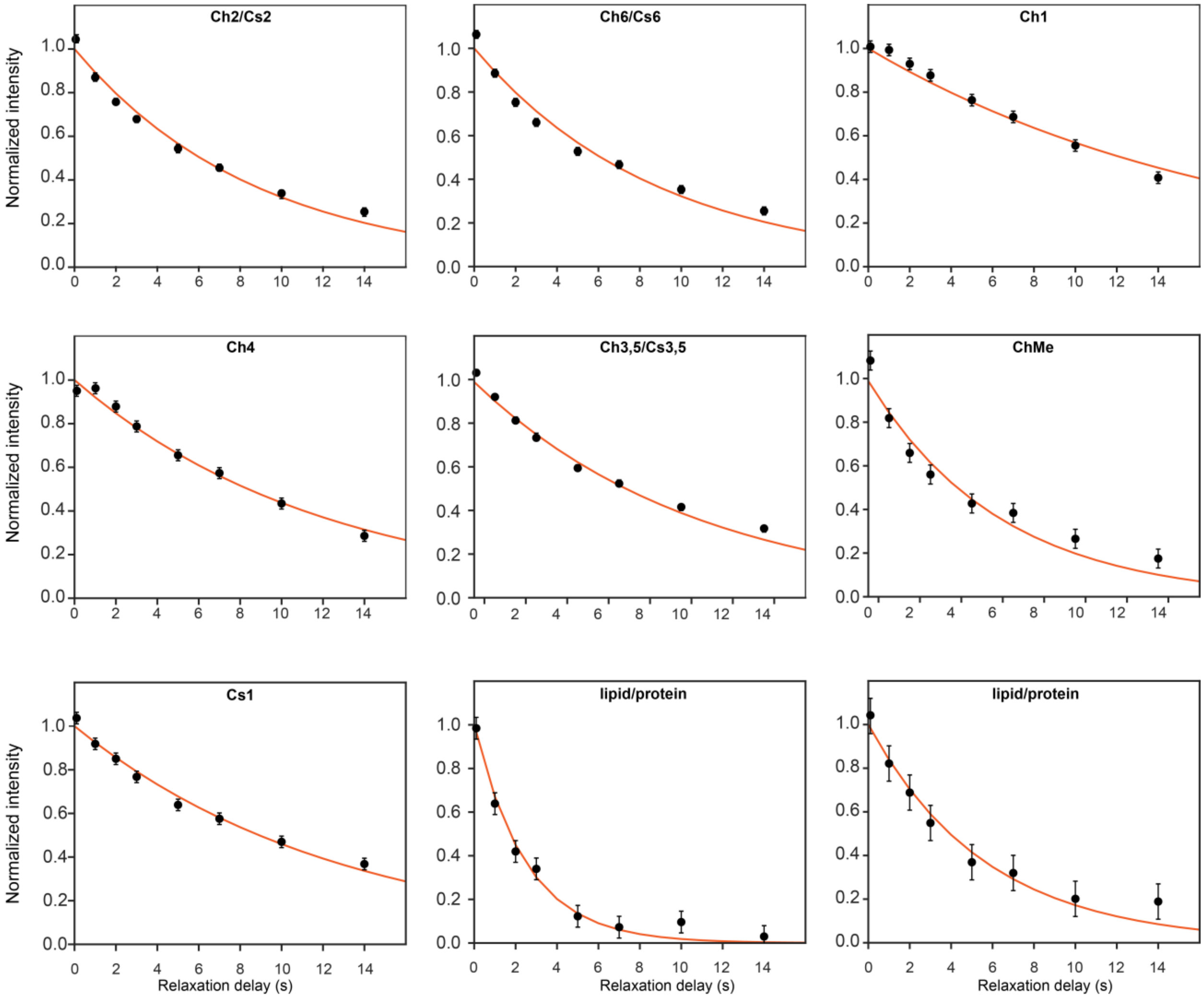

Figure S6. ^{13}C R_1 intensity decay curves of *R. delemar*. The magnetization decay during a recovery delay period π/2-τ-π/2 was measured using 2D ^{13}C T_1 hCH experiment. Intensities were extracted by integrating cross-peaks at each time point using Topspin 4.2.0 software. Measurements were performed on a Bruker Neo14.1 T (600 MHz) spectrometer at a MAS rate of 60 kHz. The dynamical parameters were determined by fitting these decay curves to a simple model free (SMF) formalism.

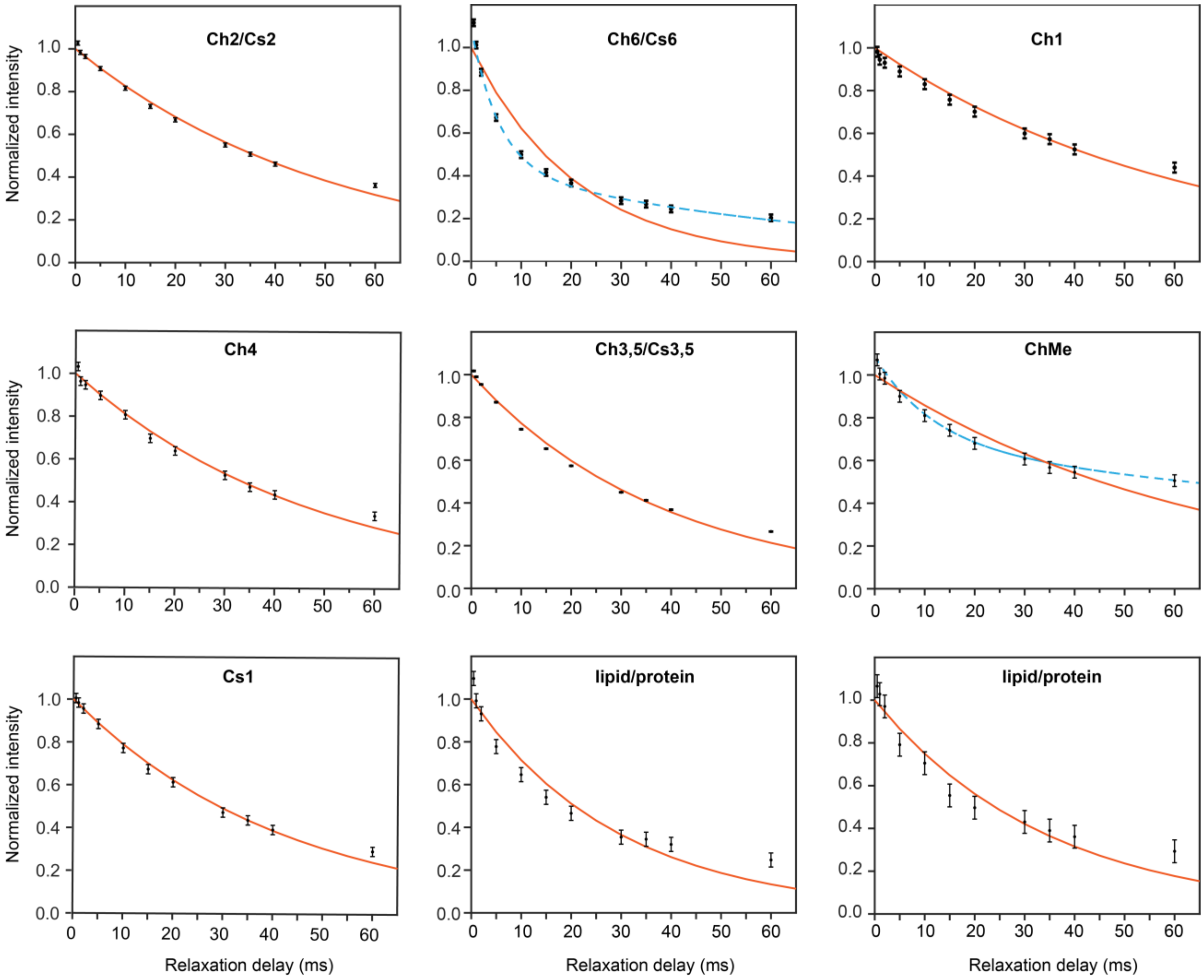

Figure S7. ^{13}C $R_{1\rho}$ intensity decay curves of *R. delemar*. The magnetization decay under spin-lock conditions was measured using 2D ^{13}C $T_{1\rho}$ hCH experiments. Intensities were extracted by integrating cross-peaks at each time point using Topspin 4.2.0 software. Measurements were performed on a Bruker Neo 600 MHz spectrometer at 60 kHz MAS with a spinlock filed strength of 17 kHz. The dynamical parameters were determined by fitting these decay curves to a simple model free (SMF) formalism. The data was fit using a single-exponential equation, except for the overlapped peak of Ch6/Cs6 and the ChMe peak with minor contributions from protein, where the fit to a double-exponential equation was also shown in dashed lines.

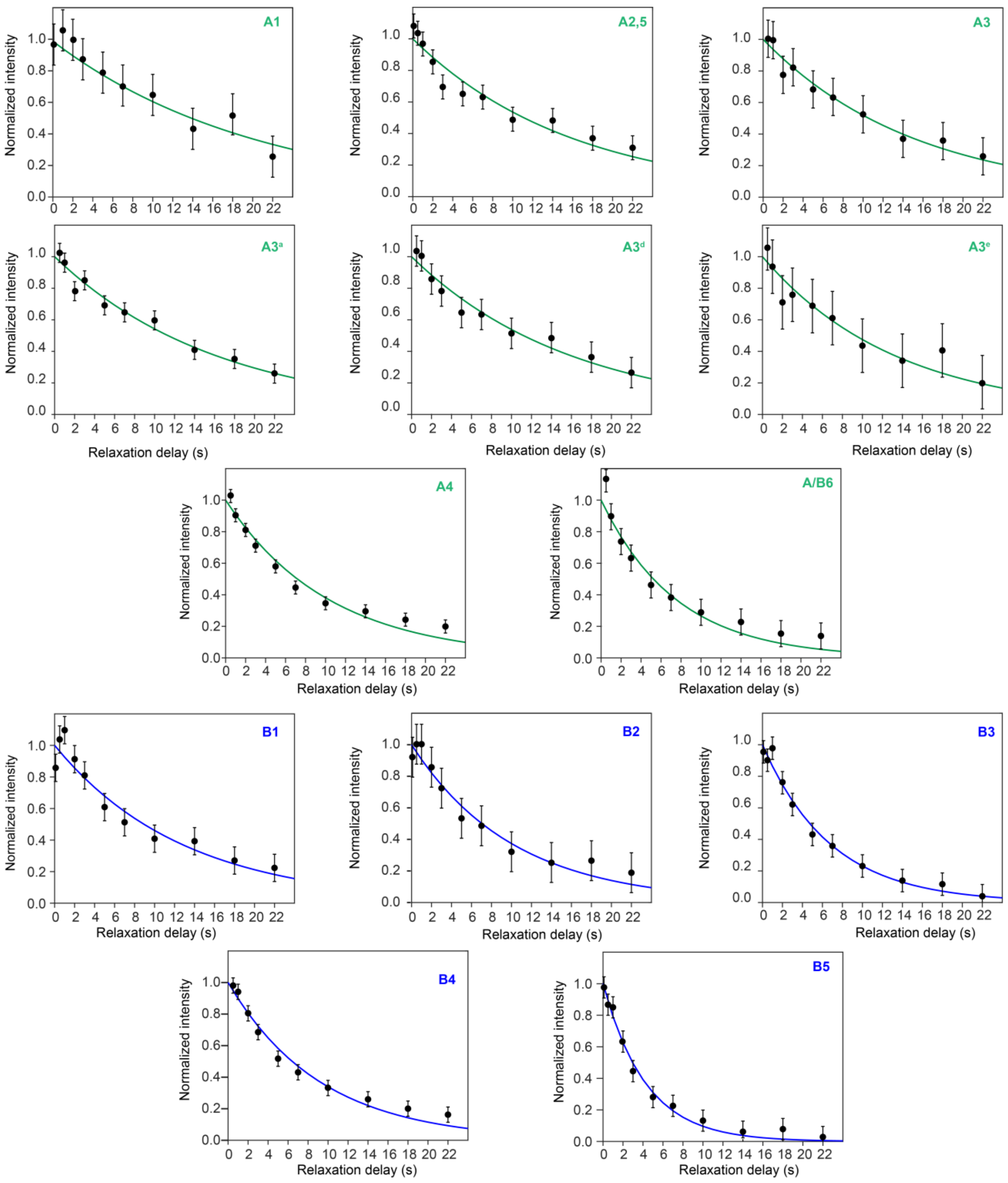

Figure S8. ^{13}C *R_1* intensity decay curves of deuterated *A. fumigatus* (RL578). The magnetization decay during a recovery delay period π/2-τ-π/2 was measured using 2D ^{13}C T_1 hCH experiment. Intensities were integrated from extracted cross-peaks at each time point using Topspin 4.1.4 software and fitted using the simple model-free (SMF) formalism. The α-glucans polymorphic forms decay intensities were extracted at the A3 site (~84 ppm) and its polymorphic forms are indicated as A3^a, A3^d, A3^e.

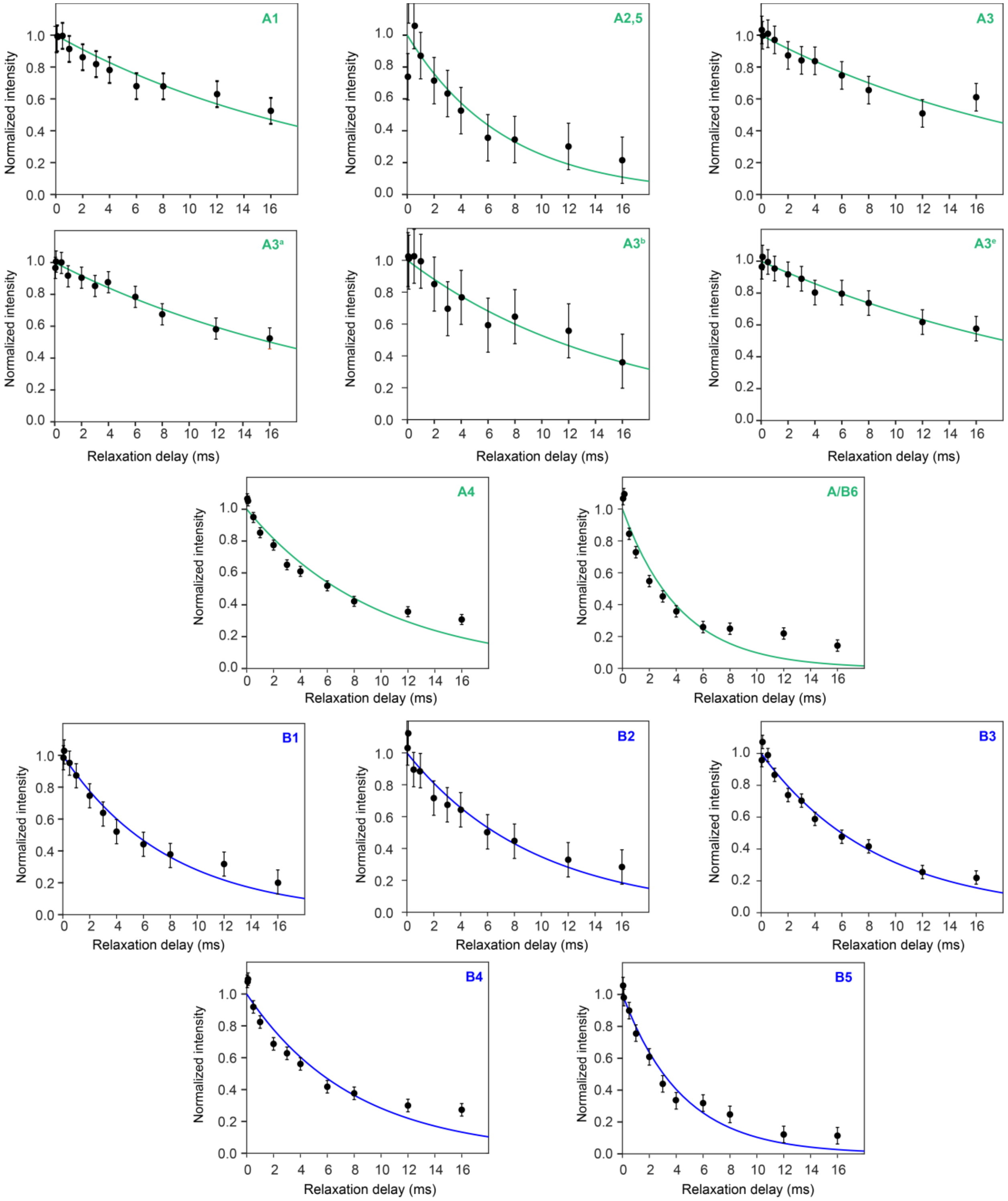

Figure S9. ^{13}C $R_{1\rho}$ intensity decay curves of deuterated *A. fumigatus* (RL578). The magnetization decay under spin-lock conditions was measured using 2D ^{13}C $T_{1\rho}$ hCH experiments. Intensities were extracted by integrating cross-peaks at each time point using Topspin 4.1.4 software. The dynamical parameters were determined by fitting these decay curves to a simple model free (SMF) formalism. The α-glucans polymorphic forms decay intensities were extracted at the A3 site (~84 ppm) and its polymorphic forms are indicated as $A3^{a}$, $A3^{d}$, $A3^{e}$.

Table S1. Experimental parameters used for *R. delemar*. The experiments for *R. delemar* were performed on 600 MHz (14.1 T) with the MAS frequency of 60 kHz (Bruker 1.3 mm MAS probe) at MSU, East Lansing. Also, on 800 MHz (18.8 T) spectrometer with the MAS frequency of 60 kHz at MagLab, Tallahassee (Home built 1.3 mm MAS probe). Also, on 800 MHz at MSU, east Lansing with the MAS frequency of 15 kHz (Phoenix 1.6 mm probe).

**Experiments**	**B_0 (T)**	**MAS (kHz)**	**CP (μs)**			**D1**	**NS**	**td2**	**td1**	**td3**	**aq2 (ms)**	**aq1 (ms)**	**aq3 (ms)**	**Water suppression**	**DIPSI-3 (ms)**	**Expt. Time (h)**
			t_{cp1}	t_{cp2}	t_{cp3}											
2D hCH	18.8	60	200 (HC-CP)	-	200 (CH-CP)	3	16	2352	256	-	19.9	4.26	-	MISSISSIPI (total duration)50 ms (rf 30 kHz)	-	2.84
2D hNH	18.8	60	2000 (HN-CP)	-	200 (NH-CP)	3	16	1306	512	-	19.9	12.8	-		-	6.82
3D coCH$_3$coNH	18.8	60	1400 (HC-CP)	8000 (CN-CP)	800 (NH-CP)	2	64	1306 (^{1}H)	32 (^{13}C)	48 (^{15}N)	19.9	5.6	4.8		-	54.6
2D c2NH$_2$	18.8	15	800 (HC-CP)	3000 (CN-CP)	2500 (NH-CP)	2.5	128	1204 (^{1}H)	128 (^{13}C)	1 (^{15}N)	19.9	3.19	-	MISSISSIPI (total duration) 250 ms	-	11.3
2D NC2	18.8	15	4000 (HN-CP)	3000 (NCCP)	-	3	128	2048 (^{13}C)	142 (^{15}N)	-	21	4.7	-	MISSISSIPI (total duration) 100 ms	-	15.1
3D hCCH TOCSY (Waltz-16)	14.1	60	200 (HC-CP)	-	200 (CH-CP)	2.5	8	4000 (^{1}H)	98 (^{13}C)	98 (^{13}C)	20	2.44	2.44		15	42.7
2D ^{1}H-^{13}C T_1 hCH	14.1	60	200 (HC-CP)	-	50 (CH-CP)	2	16	4000 (^{1}H)	256 (^{13}C)	-	20	4.26	-		-	63.8
2D ^{1}H-^{13}C $T_{1\rho}$ hCH	14.1	60	200 (HC-CP)	-	50 (CH-CP)	2	16	4000 (^{1}H)	256 (^{13}C)	-	20	4.26	-		-	22.7
2D ^{1}H-^{15}N HETCOR	18.8	15	1500 (HN-CP)	-	-	3	16	1806 (^{15}N)	256 (^{1}H)	-	29.9	4.83	-	-	-	3.41
2D ^{1}H $T_{1\rho}$ filtered ^{1}H-^{15}N HETCOR	18.8	15	2500 (HN-CP)	-	-	3	16	1806 (^{15}N)	256 (^{1}H)	-	29.9	4.83	-	-	-	3.41
1D ^{13}C CP	18.8	15	200 (HC-CP)	-	-	2.5	512	6000 (^{13}C)	-	-	30	-	-	-	-	0.35
1D ^{13}C T_1 filtered ^{1}H-^{13}C CP	18.8	15	200 (HC-CP)	-	-	2.5	512	6000 (^{13}C)	-	-	30	-	-	-	-	0.35
1D Dipolar dephasing ^{1}H-^{13}C CP	18.8	15	600 (HC-CP)	-	-	3	1024	1818 (^{13}C)	-	-	19.9	-	-	-	-	0.85

Table S2. Experimental parameters used for deuterated *A. fumigatus* (RL-578) on 600 MHz. Experiments were performed on 600 MHz (14.1 T) spectrometer equipped with 1.3 mm triple resonance MAS probe with the MAS frequency of 60 kHz.

Expt.	CP (μs)		NS	D1 (s)	td2	td1	td3	aq2 (ms)	aq1 (ms)	aq3 (ms)	DIPSI-3 (ms)	RFDR mixing (ms)	Expt. Time (h)
	t_{cp1}	t_{cp2}											
2D hCH	1600 (HC-CP)	50 (CH-CP)	16	2	1764 (^{1}H)	384 (^{13}C)	-	14.9	6.4	-	-	-	3.41
2D hCHhH (RFDR)	1600 (HC-CP)	100 (CH-CP)	16	2	1764 (^{1}H)	512 (^{13}C)	-	14.9	8.5	-	-	0.133	4.55
												0.267	4.55
												0.8	4.55
3D hCCH TOCSY (Waltz-16)	1600 (HC-CP)	100 (CH-CP)	8	2	1764 (^{1}H)	116 (^{13}C)	116 (^{13}C)	14.9	1.93	1.93	15	-	59.80
1D ^{13}C CP	1400 (HC-CP)	-	512	2	1818	-	-	19.9	-	-	-	-	0.28

Table S3. Experimental parameters used for protonated and deuterated *A. fumigatus* samples (RL-578) on 800 MHz. Experiments were performed on 800 MHz (18.8 T) spectrometer equipped with 1.6 mm Phoenix MAS probe with the MAS frequency of 40 kHz.

Expt.	CP (μs)		NS	D1 (s)	td2	td1	td3	aq2 (ms)	aq1 (ms)	aq3 (ms)	DIPSI-3 (ms)	RFDR mixing (ms)	Expt. Time (h)
	t_{cp1}	t_{cp2}											
2D hCH	1000 (HC-CP)	50 (CH-CP)	16	3	1204 (^{1}H)	512 (^{13}C)	-	19.9	6.4	-	-	-	6.82

Table S4. Experimental parameters used for *C. albicans* (JKC2830). The experiments for *C. albicans* were performed on 800 MHz (18.8 T) spectrometer with the MAS frequency of 15 kHz.

Experiments	MAS	D1	NS	td2	td1	td3	aq2 (ms)	aq1 (ms)	aq3 (ms)	Decoupling/ Water suppression	*J*-evolution (ms)	DIPSI-3 (ms)	Expt. Time (h)
2D *J*-INEPT-HSQC	15	2	16	8000 (^1H)	768 (^{13}C)	-	40	9.6	-	SPINAL-64 (rf 71.429 kHz) WALTZ-16 (rf 10 kHz) MISSISSIPI (total duration) 40 ms (rf 25.994 kHz	2 (τ_1) 2 (τ_2	-	6.82
2D hCCH TOCSY (DIPSI-3)	15	1.89	8	2614 (^1H)	512 (^{13}C)	1	39.9	2.56	-		1.78 (τ_1) 1.19 (τ_2	25.5	2.15
3D hCCH TOCSY (DIPSI-3)	15	1.89	8	2614 (^1H)	128 (^{13}C)	128	39.9	2.56	2.56				68.8

Table S5. ^{1}H and ^{13}C chemical shift of rigid carbohydrates of *R. delemar*.

	Carbohydrates	C1	C2	C3	C4	C5	C6	CH_3	^{15}N	Reference
Rigid	Chitin (Ch)	104.2<br>4.6	55.4<br>3.7	74.1<br>3.6	83.3<br>3.4	75.7<br>3.6	60.7<br>3.7	23.7<br>1.9	123.7<br>8.5	Kang *et al.* 2018[7]<br>Fernando *et al.* 2021[8]<br>Cheng *et al.* 2024[9]
	Chitosan (Cs)	99.5<br>4.9	55.4<br>3.7	71.6<br>3.8	80<br>4.3	75<br>3.6	60.7<br>3.7	-	33.6<br>5.0	
	β-1,3-glucan (B)	104.2<br>3.8	74.4<br>3.6	86.8<br>3.4	68.2<br>3.3	77.4<br>3.4	61.3<br>3.7	-	-	

Table S6. ^{1}H and ^{13}C chemical shifts of deuterated *A. fumigatus* (RL-578).

	Carbohydrates	Type	C1	C2	C3	C4	C5	C6	Reference
Rigid	β-1,3-glucan (B)	/	103.6<br>5.1	74.1<br>3.8	86.6<br>3.8	68.2<br>3.67	77.3<br>3.5	61.1<br>4.1	Dickwella Widanage et al.[10]
	α-1,3-glucan (A)	a	100.8<br>5.6	71.6<br>4.55	84.3<br>4.5	69.3<br>4.1	71.6<br>4.55	61.1<br>4.07	
		d	100.8<br>5.44	71.5<br>4.1	84.6<br>4.1	69.3<br>4.1	71.5<br>4.1	61.1<br>4.07	
		e	100.7<br>5.23	71.5<br>3.73	84.7<br>3.5	69.3<br>4.1	71.5<br>3.73	61.1<br>4.07	

Table S7. ^{1}H and ^{13}C chemical shift of rigid carbohydrates of *C. albicans* (JKC2830).

Carbohydrates	forms	C1	C2	C3	C4	C5	C6	Reference
β-1,3-glucan (B)	a	103.6<br>4.56	74.1<br>3.34	85.4<br>4.1	69.7<br>4.20/3.87	76.0<br>3.62	61.6<br>3.75, 3.93	Shim *et al.* 2007[11]<br>Fairweather *et al.* 2009[12]<br>Saito *et al.* 1979[13]
	b	---	74.40	84.75<br>4.13	70.37	---	61.79<br>3.80, 3.90	
	c	---	74.62<br>---	86.44<br>4.28	71.27<br>---	---	62.40<br>3.80, 3.90	Lowman *et al.* 2011[14]
β-1,3,6-glucan (Br)		103.36<br>4.47	75.09<br>3.48	85.33<br>3.75	---	---	68.74<br>---	
β-1,6-glucan (H)	a	102.72<br>4.58	74.03<br>3.35	76.69<br>3.47	70.31<br>3.45	---	---	
	b	103.71<br>4.52	74.01<br>3.35	76.63<br>3.51	70.25<br>3.45	---	---	
	c	103.39<br>4.46	74.01<br>3.35	76.30<br>3.45	70.30<br>3.45	---	---	
α-1,6-Mannan ($Mn^{1,6}$)		102.98<br>5.13	71.00<br>4.00/3.40	74.10<br>3.34	67.70<br>3.68	71.00<br>4.00/3.40	---	Latge *et al.* 1994[15]<br>Chakraborty *et al.* 2021[16]<br>Kuraoka *et al.* 2021[17]
α-1,2-Mannan ($Mn^{1,2}$)	a	101.28<br>5.27	79.10<br>4.10	70.90<br>4.00	67.80<br>3.70	74.00<br>3.70	61.80<br>3.80/3.90	
	b	98.66<br>5.11	79.42<br>4.00	70.89<br>3.40	67.00<br>3.80	73.40<br>3.75	61.37<br>3.70/ 3.80	Kuraoka *et al.* 2021[17]<br>Kuraoka *et al.* 2018[18]
	c	100.72<br>5.15	78.20<br>4.10	70.30<br>3.90	68.00<br>3.60	74.00<br>3.70	61.40<br>3.80, 3.90	
	d	101.40<br>5.38	79.10<br>4.10	70.90<br>4.00	67.90<br>3.70	73.40<br>3.60	61.80<br>3.80/ 3.90	
	e	102.87<br>5.05	78.90<br>3.93	70.70<br>3.40	66.90<br>3.80	74.00<br>3.30	61.90<br>3.80/ 3.90	
	f	101.33<br>5.27	79.10<br>4.10	71.00<br>3.95	67.90<br>3.71	74.20<br>3.78	62.10<br>3.80,3.90	
	g	100.50<br>5.36	78.50<br>4.27	70.40<br>4.20	68.00<br>3.63	73.50<br>3.80	61.50<br>3.80, 3.90	
	h	102.90<br>5.04	78.90<br>3.93	70.20<br>4.20	66.60<br>3.90	73.60<br>3.80	61.84<br>3.80, 3.90	
Galactose/Glucose or their derivatives (Gl)	a	90.00<br>5.90	---	74.30<br>4.30	70.30<br>4.20	--	61.70<br>3.80/ 3.90	Fontaine *et al.* 2011[19]
	c	94.52<br>4.90	72.50<br>3.80	73.60<br>3.60	67.40<br>3.60	---	61.87<br>3.70, 3.80	Archbald et al. 1981[20]<br>Fontaine *et al.* 2011[19]
	d	94.95<br>5.18	72.50<br>3.90	71.30<br>3.85	67.60<br>3.66		61.90<br>3.70, 3.80	
	e	89.26<br>6.06	---	74.50<br>4.30	71.40<br>4.40	---	62.50<br>3.80, 3.90	
Glucose (Glc)	a (α)	92.94<br>5.20	72.40<br>3.50	72.40<br>3.50	70.60<br>3.40	---	61.56<br>3.70, 3.80	Archbald *et al.* 1981[20]
	b (β)	96.84<br>4.63	74.90<br>3.25	76.60<br>3.50	70.50<br>3.40	---	61.79<br>3.70, 3.90	
Unk1		99.60<br>4.92	72.67<br>3.50	---	70.70<br>3.40	67.00<br>3.40	61.55<br>3.70/3.80	
Unk2		100.94<br>4.86	73.30<br>3.60	---	71.00<br>3.88/3.40	67.60<br>3.70	61.88<br>3.70/3.80	

Table S8. Dynamical parameters of *R. delemar* extracted from SMF formalism. Ch and Cs stands for chitin and chitosan resonances respectively.

Carbon number	^{13}C R_1 (s^{-1})	^{13}C $R_{1\rho}$ (s^{-1})	S^2	$\tau_{c,eff}$ (ns)	χ^2
Ch1	0.056±0.003	16.08±0.86	0.92±0.03	11.6±0.6	3.03
Ch/Cs2	0.113±0.003	19.16±0.33	0.87±0.02	8.9±0.2	10.9
Ch/Cs3/5	0.091±0.001	25.78±0.13	0.87±0.01	11.6±0.1	107
Ch4	0.0826±0.005	20.82±0.80	0.88±0.04	10.9±0.4	2.62
Ch/Cs6	0.113±0.003	47.39±1.45	0.79±0.01	14.1±0.3	42.1
Ch Me	0.156±0.014	15.33±0.89	0.86±0.01	6.7±0.4	5.40
Cs1	0.077±0.004	23.57±0.85	0.88±0.04	12.1±0.4	1.80
Protein/lipid at 30 ppm	0.401±0.038	33.58±1.93	0.67±0.01	6.2±0.3	4.45
Protein/lipid at 40 ppm	0.176±0.029	29.07±2.55	0.79±0.01	8.9±0.9	1.89

Table S9. Dynamical parameters of *A. fumigatus* (RL578) extracted from SMF formalism.

Carbon number	^{13}C R_1 (s^{-1})	^{13}C $R_{1\rho}$ (s^{-1})	S^2	$\tau_{c,eff}$ (ns)	χ^2
B1	0.077±0.012	12.61±1.72	0.92±0.01	8.9±1.1	1.33
B2	0.098±0.019	10.55±2.14	0.91±0.01	7.1±1.1	0.6
B3	0.146±0.016	11.58±0.78	0.88±0.01	6.0±0.4	1.43
B4	0.108±0.009	12.62±0.95	0.89±0.01	7.39±0.45	4.62
B5	0.234±0.028	22.63±2.33	0.79±0.02	6.7±0.6	1.57
B/A6	0.132±0.034	23.26±1.98	0.85±0.02	9.4±1.57	6.87
A1	0.048±0.009	4.71±0.86	0.96±0.01	6.8±0.9	0.90
A2/5	0.062±0.008	13.85±3.64	0.92±0.01	10.2±1.5	1.51
A3[a]	0.061±0.005	4.32±0.63	0.95±0.01	5.67±0.52	0.69
A3[d]	0.074±0.022	6.39±2.13	0.94±0.01	6.4±1.62	0.34
A3[e]	0.061±0.010	3.80±0.63	0.95±0.01	5.29±0.63	0.38
A4	0.096±0.008	10.23±0.71	0.91±0.01	7.0±0.4	5.28

Supplementary References

1. Lamley, J. M.; Lougher, M. J.; Sass, H. J.; Rogowski, M.; Grzesiek, S.; Lewandowski, J. R., Unraveling the complexity of protein backbone dynamics with combined 13C and 15N solid-state NMR relaxation measurements. *Phys. Chem. Chem. Phys.* **2015,** *17* (34), 21997-22008.
2. Yarava, J. R.; Orwick-Rydmark, M.; Ryoo, D.; Hofstetter, A.; Gumbart, J. C.; Habeck, M.; van Rossum, B.-J.; Linke, D.; Oschkinat, H., Probing the Dynamics of Yersinia Adhesin A (YadA) in Outer Membranes Hints at Requirements for β-Barrel Membrane Insertion. *J. Am. Chem. Soc.* **2025**.
3. Busi, B.; Yarava, J. R.; Hofstetter, A.; Salvi, N.; Cala-De Paepe, D.; Lewandowski, J. R.; Blackledge, M.; Emsley, L., Probing Protein Dynamics Using Multifield Variable Temperature NMR Relaxation and Molecular Dynamics Simulation. *J. Phys. Chem. B* **2018,** *122* (42), 9697-9702.
4. Yates, J. R.; Pham, C. J.; Pickard, C. J.; Mauri, F.; Amado, A. M.; Gil, A. M.; Brown, S. P., An Investigation of Weak CH···O Hydrogen Bonds in Maltose Anomers by a Combination of Calculation and Experimental Solid-State NMR Spectroscopy. *J. Am. Chem. Soc.* **2005,** *127*, 10216-10220.
5. Lipari, G.; Szabo, A., Model-free approach to the interpretation of nuclear magnetic resonance relaxation in macromolecules. 1. Theory and range of validity. *J. Am. Chem. Soc.* **1982,** *104*, 4546-4559.
6. Lipari, G.; Szabo, A., Model-free approach to the interpretation of nuclear magnetic resonance relaxation in macromolecules. 2. Analysis of experimental results. *J. Am. Chem. Soc.* **1982,** *104*, 4559-4570.
7. Kang, X.; Kirui, A.; Muszynski, A.; Dickwella Widanage, M. C.; Chen, A.; Azadi, P.; Wang, P.; Mentink-Vigier, F.; Wang, T., Molecular architecture of fungal cell walls revealed by solid-state NMR. *Nat. Commun.* **2018,** *9*, 2747.
8. Fernando, L. D.; Dickwella Widanage, M. C.; Penfield, J.; Lipton, A. S.; Washton, N.; Latgé, J.-P.; Wang, P.; Zhang, L.; Wang, T., Structural Polymorphism of Chitin and Chitosan in Fungal Cell Walls From Solid-State NMR and Principal Component Analysis. *Front. Mol. Biosci.* **2021,** *8*.
9. Cheng, Q.; Dickwella Widanage, M. C.; Yarava, J. R.; Ankur, A.; Latgé, J.-P.; Wang, P.; Wang, T., Molecular architecture of chitin and chitosan-dominated cell walls in zygomycetous fungal pathogens by solid-state NMR. *Nat. Commun.* **2024,** *15*, 8295.
10. Dickwella Widanage, M. C.; Gautam, I.; Sarkar, D.; Mentink-Vigier, F.; Vermaas, J. V.; Ding, S.-Y.; Lipton, A. S.; Fontaine, T.; Latgé, J.-P.; Wang, P.; Wang, T., Adaptative survival of Aspergillus fumigatus to echinocandins arises from cell wall remodeling beyond β−1,3-glucan synthesis inhibition. *Nat. Commun.* **2024,** *15* (1), 6382.
11. Shim, J. H.; Sung, K. J.; Cho, M. C.; Choi, W. A.; Yang, Y.; Lim, J. S.; Yoon, D. Y., Antitumor Effect of Soluble b-1, 3-Glucan from Agrobacterium sp. R259 KCTC 1019. *J. Microbiol. Biotechnol.* **2007,** *17* (9), 1513-1520.
12. Fairweather, J. K.; Him, J. L. K.; Heux, L.; Driguez, H.; Bulone, V., Structural characterization by ^{13}C-NMR spectroscopy of products synthesized in vitro by polysaccharide synthases using ^{13}C-enriched glycosyl donors: application to a UDP-glucose:(1→ 3)-b-d-glucan synthase from blackberry (Rubus fruticosus). *Glycobiology* **2004,** *14* (9), 775-781.
13. Saitô, H.; Ohki, T.; Sasaki, T., A 13C-nuclear magnetic resonance study of polysaccharide gels. Molecular architecture in the gels consisting of fungal, branched (1→ 3)-b-D-glucans (lentinan and schizophyllan) as manifested by conformational changes induced by sodium hydroxide. *Carbohydr. Res.* **1979,** *74* (1), 227-240.

14. Lowman, D. W.; West, L. J.; Bearden, D. W.; Wempe, M. F.; Power, T. D.; Ensley, H. E.; Haynes, K.; Williams, D. L.; Kruppa, M. D., New Insights into the Structure of (1→3,1→6)-β-D-Glucan Side Chains in the Candida glabrata Cell Wall. *PLoS One* **2011,** *6*, e27614.

15. Latge, J. P.; Kobayashi, H.; Debeaupuis, J. P.; Diaquin, M.; Sarfati, J.; Wieruszeski, J. M.; Parra, E.; Bouchara, J. P.; Fournet, B., Chemical and immunological characterization of the extracellular galactomannan of Aspergillus fumigatus. *Infect. Immun.* **1994,** *62* (12), 5424-5433.

16. Chakraborty, A.; Fernando, L. D.; Fang, W.; Dickwella Widanage, M. C.; Wei, P.; Jin, C.; Fontaine, T.; Latgé, J. P.; Wang, T., A molecular vision of fungal cell wall organization by functional genomics and solid-state NMR. *Nat. Commun.* **2021,** *12*, 6346.

17. Kuraoka, T.; Yamada, T.; Takatsutsumi, Y.; Ogawa, Y.; Kobayashi, H., Anomeric Proton and Carbon (H1-C1) NMR Chemical Shifts of Antigenic Mannans Obtained from Pathogenic Yeast Candida tropicalis. *Adv. Microbiol.* **2021,** *11*, 296-301.

18. Kuraoka, T.; Ishiyama, A.; Oyamada, H.; Ogawa, Y.; Kobayashi, H., Presence of O-glycosidically linked oligosaccharides in the cell wall mannan of Candida krusei purified with Benanomicin A. *FEBS Open Bio.* **2018,** *9*, 129-136.

19. Fontaine, T.; Delangle, A.; Simenel, C.; Coddeville, B.; van Vliet, S. J.; van Kooyk, Y.; Bozza, S.; Moretti, S.; Schwarz, F.; Trichot, C.; Aebi, M.; Delepierre, M.; Elbim, C.; Romani, L.; Latgé, J. P., Galactosaminogalactan, a New Immunosuppressive Polysaccharide of Aspergillus fumigatus. *PLoS Pathog.* **2011,** *7*, e1002372.

20. Archbald, P. J.; Fenn, M. D.; Roy, A. B., 13C-N.M.R. studies of D-glucose and D-galactose monosulphates. *Carbohydr. Res.* **1981,** *93*, 177-190.
